# An adolescent patient with anti-N-methyl-D-aspartate receptor encephalitis with motor aphasia as the first symptom and complicated by peripheral nerve damage: A case report and literature review

**DOI:** 10.1097/MD.0000000000042436

**Published:** 2025-05-09

**Authors:** Lei Shen, Hanxing Liu, Xi Liu, Lei Zhang, Jin Wang, Niao Yang, Nao Yan

**Affiliations:** a Department of Neurosurgery, Zhongnan Hospital of Wuhan University, Wuhan, China; b Department of Neurology, Zhongnan Hospital of Wuhan University, Wuhan, China; c Department of Cardiology, Wuhan University of Science & Technology, Hanyang Hospital, Wuhan, China.

**Keywords:** anti-N-Methyl-D-Aspartate, aphasia, case report, encephalitis, NMDA, peripheral nerve damage

## Abstract

**Rationale:**

Anti-N-methyl-D-aspartate receptor (anti-NMDAR) encephalitis is a type of autoimmune encephalitis, and the common first symptoms are mental disorders, seizures, and rarely aphasia in patients. Meanwhile, movement disorders associated with anti-NMDAR encephalitis are usually chorea and dystonia, with peripheral nerve damage being rare.

**Patient concerns:**

We present a case of anti-NMDAR encephalitis with motor aphasia as the first symptom. The patient, a 16-year-old female, was admitted to the hospital with further progression of the disease, complicated by grand mal seizures with peripheral nerve damage.

**Diagnoses:**

Anti-NMDAR encephalitis.

**Interventions:**

The patient accepted first-line therapy, including methylprednisolone and intravenous immunoglobulin shock therapy, rituximab second-line treatment (rituximab), and third-line therapies (mycophenolate mofetil), as well as efgartigimod as an additional therapy.

**Outcomes:**

After 6 weeks of comprehensive treatment, the patient’s muscle strength in both lower limbs recovered, and the psychiatric symptoms and seizures improved.

**Lessons:**

This case broadens the range of clinical symptoms of anti-NMDAR encephalitis, and we should recognize that motor aphasia may also be one of the first symptoms in adolescent patients with anti-NMDAR encephalitis. What’s more, efgartigimod may be a promising treatment for patients with anti-NMDAR encephalitis.

## 1. Introduction

Anti-N-methyl-D-aspartate Receptor (anti-NMDAR) encephalitis is an immune-mediated inflammatory disease of the central nervous system, prevalent in adolescents and children.^[1,2]^ The currently accepted pathogenesis of anti-NMDAR encephalitis is that excessive antibodies against the NMDAR present in the patients’ central nervous system and attack the NMDAR on the postsynaptic membrane, resulting in severe disorders of synaptic plasticity and NMDAR signaling.^[3,4]^ As a result of impaired NMDAR function, patients usually present first with symptoms such as mental disorders, behavioral changes, and seizures, with studies suggesting that approximately 90% of patients have significant psychiatric or behavioral symptoms.^[5,6]^ However, aphasia, especially motor aphasia, is less common.^[7,8]^ Meanwhile, motor disorders complicating anti-NMDAR encephalitis are usually stereotyped movements, chorea, and dystonia, and peripheral nerve damage and muscle weakness are rare.^[9–11]^

Anti-NMDAR encephalitis is considered a curable autoimmune encephalitis.^[12,13]^ The current mainstream treatment for anti-NMDAR encephalitis is immunotherapy, which specifically includes first-line immunotherapy (steroids, intravenous immunoglobulin [IVIG], plasmapheresis), second-line immunotherapy (rituximab, cyclophosphamide), and further/third-line immunotherapy (mycophenolate mofetil, methotrexate, and bortezomib). However, much controversy still surrounds the treatment of anti-NMDAR encephalitis, and the dosing regimen and long-term outcomes of immunotherapy have not yet been determined.^[14,15]^

In order to recognize the anti-NMDAR encephalitis early in terms of clinical symptoms, here we report a case of an adolescent patient with anti-NMDAR encephalitis with motor aphasia as the first symptom.

## 2. Case report

In late December 2023, a 16-year-old female presented to our hospital with motor aphasia for 6 days and 1 seizure, without limb weakness or mental disorders. 1 week ago, the patient presented with cold symptoms including cough and fever. 6 days ago, the patient presented with aphasia symptoms such as poor fluency in speech and difficulty in choosing words in school, which she did not seek medical attention for at that time. One day ago, the patient had a seizure with bruising of the face and stiffness of the limbs, which lasted for several minutes and then resolved. The patient was then admitted to the intensive care unit (ICU) of the Department of Neurology of our hospital for treatment. We performed a detailed neurological physical examination (PE), which showed that the patient did not have any positive signs and symptoms other than motor aphasia with no abnormalities on PE.

**Figure 1.**
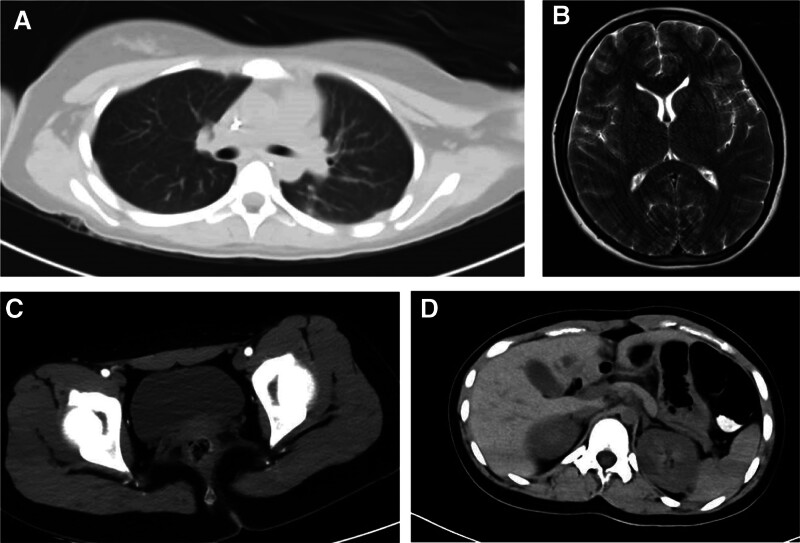
Imaging of the patient after admission to the hospital. All imaging tests found no suspicious tumors. (A) The result of the chest CT scan. (B) The result of the brain MRI. (C) The result of the pelvis CT scan. (D) The result of the abdomen CT scan.

**Figure 2.**
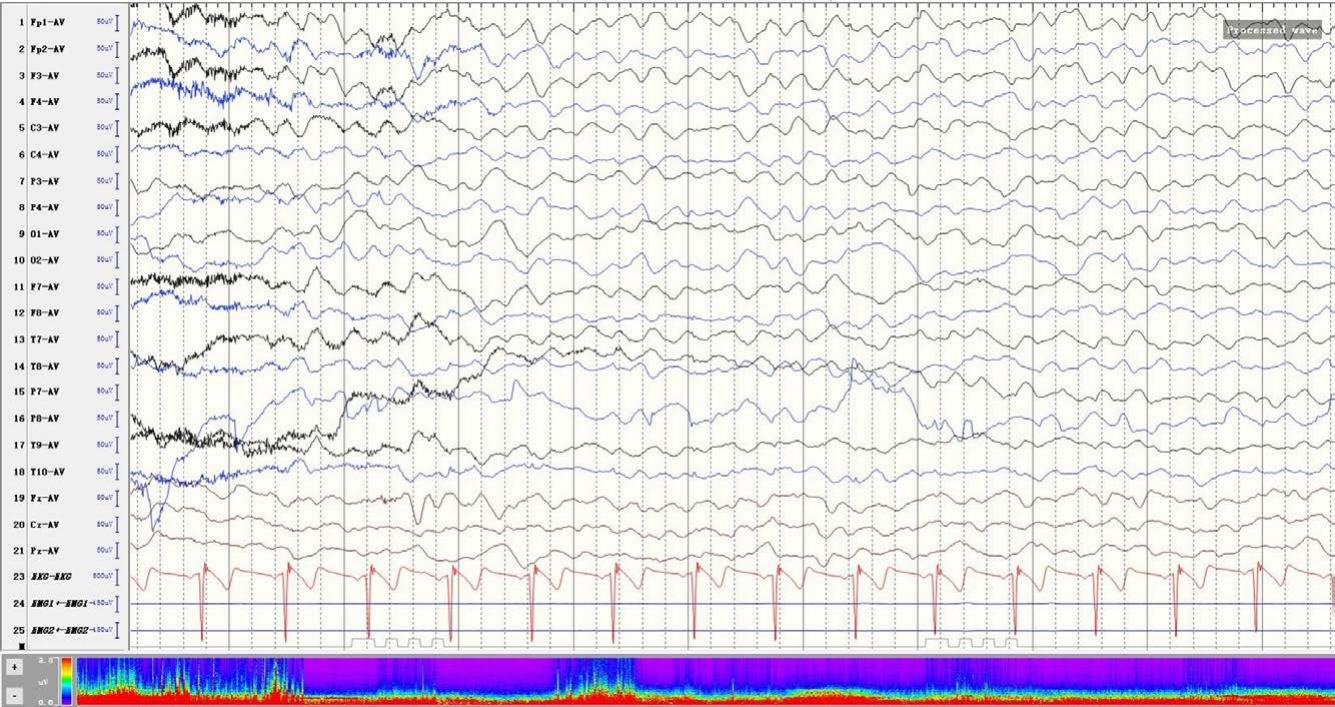
The EEG results of the patient during a seizure. The patient’s EEG rhythms were severely dysregulated, with epileptiform activity. EEG = electroencephalogram.

**Figure 3.**
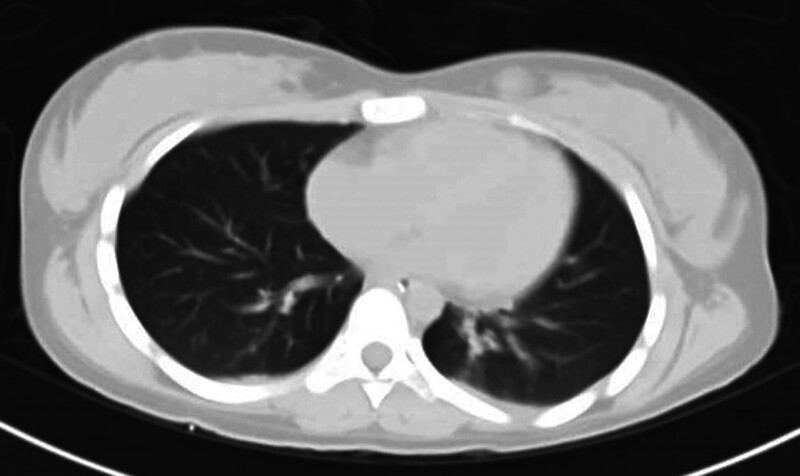
The chest CT scan after the patient developed a fever. The CT scan showed that she had pneumonia.

On the second day of admission, the patient developed psychiatric symptoms and manic episodes. The examination results of the lumbar puncture suggested that the titer of anti-NMDAR antibody in the cerebrospinal fluid (CSF) was 1:10, and the nucleated cells increased, with a normal protein level in CSF. The patient was diagnosed with anti-NMDAR encephalitis. MRI of the brain showed no obvious abnormality, and CT of the chest, abdomen, and pelvis did not find any tumor (Fig. 1A–D). The patient immediately accepted first-line therapy, including methylprednisolone (800 mg/day) and IVIG shock therapy (17.5 g/day). She was still manic and had intermittent focal seizures. At the time of the patient’s seizure, she accepted an electroencephalogram (EEG) test. EEG results showed that the patient’s EEG background was diffuse slow waves, with asynchronous 2 Hz biphasic waves, triphasic waves, and a few spike waves in the bilateral frontal leads (Fig. 2), and then she accepted quetiapine (100 mg/day), depakene (1000 mg/day), and dexmedetomidine (200 ug once for manic episode) for symptomatic treatment. At 1 week of admission, the patient developed a fever, and the lung CT showed that she had pneumonia (Fig. 3), and she was given sulperazon (6 g/day) to control the infection. After 2 days of antibiotic treatment, her temperature returned to normal, and a repeat blood test showed that the white blood cell count had dropped from 14.8*10^9^/L to 5.12*10^9^/L. 2 weeks after admission, the patient’s condition had not improved significantly, and the results of the blood lymphocyte subpopulation examination indicated that the proportion of B cells was as high as 30.8%, and rituximab was added as a second-line treatment while maintaining the original drug regimen. After the use of rituximab, the patient’s mania improved, and the antipsychotic drugs were reduced.

**Figure 4.**
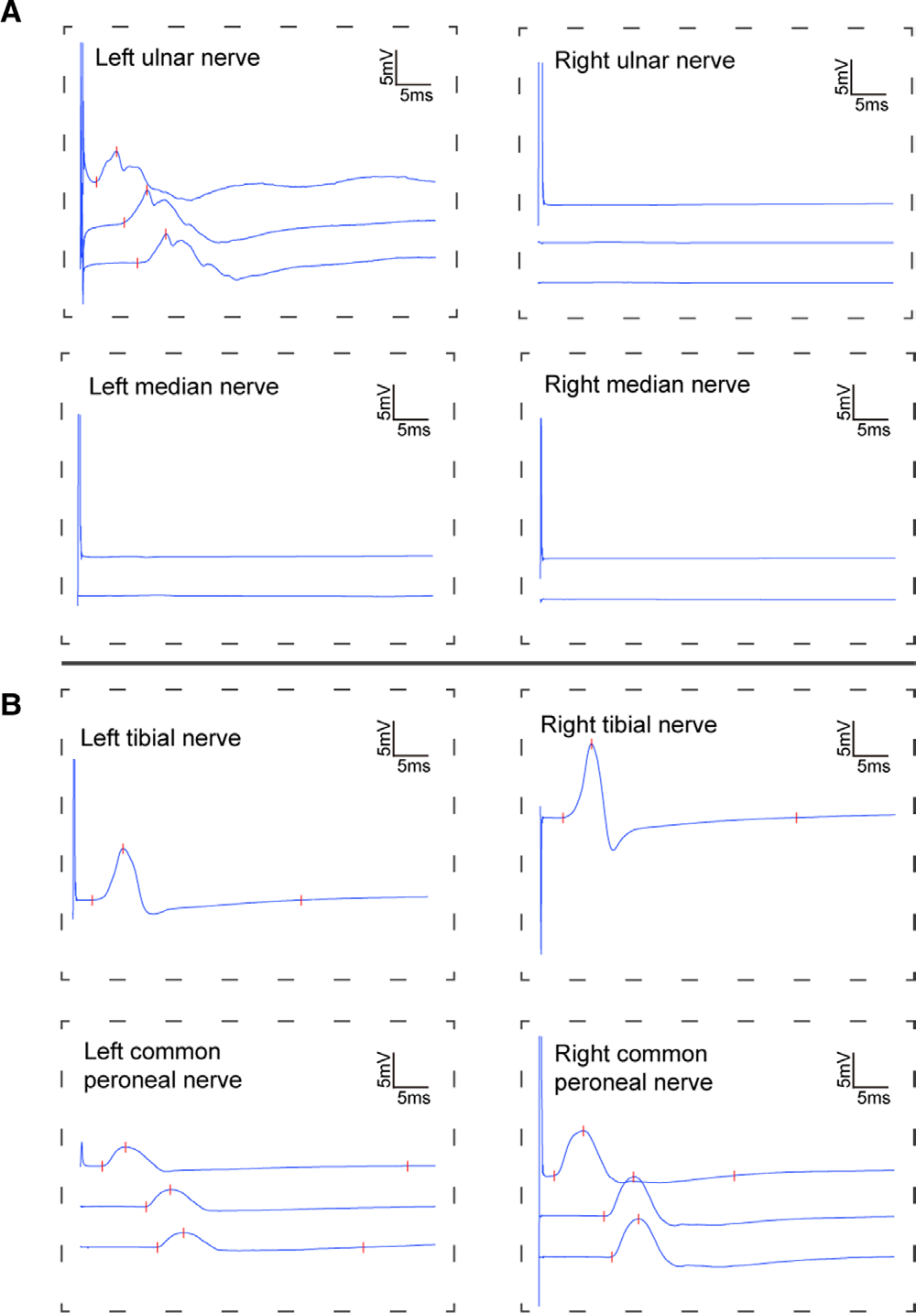
The electromyography results of the patient after transfered from the ICU. The electromyography showed peripheral nerve damage in both upper and lower limbs. (A) The electromyographic findings of bilateral ulnar and median nerves. (B) The electromyographic findings of bilateral tibial and common peroneal nerves. ICU = intensive care unit.

At 4 weeks of admission, the patient’s symptoms were relieved, she could speak simple words and cooperate to complete simple movements, antipsychotic drugs were stopped, and she was transferred from the ICU. The next day, a PE demonstrated that the muscle strength of her limbs was grade 3, tendon reflexes were reduced, and muscle tone was flaccid. Electromyography showed peripheral nerve damage in both upper and lower limbs, with more severe axonal damage in both upper limbs (Fig. 4). We considered that the patient might have a complication of autoimmune peripheral neuropathy and performed another lumbar puncture to examine for peripheral neuropathy antibodies. We checked for 12 peripheral neuropathy antibodies including anti-Sulfatide, anti-GM1, anti-GM2, anti-GM3, anti-GM4, anti-GD1a, anti-GD1b, anti-GD2, anti-GD3, anti-GT1a, anti-GT1b, and anti-GQ1b, however the results were all negative. Considering that the patient had peripheral nerve damage, after intradepartmental discussions and communicating with the family, we decided to treat the patient with Efgartigimod. After 2 days of treatment with Efgartigimod (400 mg/d), the patient’s muscle strength of both lower limbs recovered to grade 5, the muscle strength of both proximal upper limbs was grade 4, and the muscle strength of both distal upper limbs was still grade 3. 6 weeks after admission, the CSF was reexamined, and the titer of the anti-NMDAR antibody had decreased from 1:10 to 1:1. Long-term immunotherapy with mycophenolate mofetil (1 g/day) was added on top of steroids, and the patient was discharged after rehabilitation.

## 3. Literature review

Fewer patients with anti-NMDAR receptor encephalitis develop aphasia during the course of the disease. We conducted a systematic literature search to provide an overview of the demographics, clinical characteristics, and outcomes of patients with anti-NMDAR encephalitis with aphasia. Therefore, we searched the medical database PUBMED on February 28, 2024 using the terms “aphasia” and “NMDAR” and additionally checked the respective reference lists. We considered articles published since January 2005 that were written in English. The results are summarized in Table 1.

**Table 1:** Clinical characteristics of the anti-NMDAR encephalitis patients with aphasia.

Authors	Years	Gender	Age	First Symptoms	Tumor	Treatment	Prognosis
Vasilios et al^[7]^	2018	Female	29	motor aphasia	No	First-line and second-line treatments	Rehabilitation
Kumaran et al^[8]^	2014	Female	4	Seizures	No	First-line and second-line treatments	Rehabilitation
Carsten et al^[16]^	2014	Male	67	Hemianopsia, aphasia and right hemiparesis	No	First-line and second-line treatments	Clinical improvement
Nozomi et al^[17]^	2018	Female	31	Mental disorders	Yes	First-line treatment and tumor resection	Clinical improvement
Alana et al^[18]^	2014	Female	7	Behavioral disorders	No	First-line and second-line treatments	Rehabilitation
		Female	8	Seizures	No	First-line treatment	Clinical improvement
		Female	14	Seizures	No	First-line and second-line treatments	Clinical improvement
Julien et al^[19]^	2018	Female	29	motor aphasia	Yes	First-line treatment and tumor resection	Rehabilitation

Anti-NMDAR = anti-N-methyl-D-aspartate receptor.

Previous studies reported 8 anti-NMDAR patients with aphasia, including 7 female patients, and 1 male patient, ranging in age from 4 to 67 years old. 3 patients had aphasia as a first symptom (all adults), 5 developed aphasias during the course of the disease, and no adolescents or children started with aphasia as a first symptom. All patients received first-line treatment with steroids and IVIG, 5 patients received second-line treatment including rituximab, mycophenolate mofetil, and cyclophosphamide, and 2 patients underwent ovarian tumor resection surgery after the discovery of ovarian tumors. 4 of these patients recovered completely, 2 had clinical improvement, and 1 patient developed long-term speech function and memory deficits.

## 4. Discussion

The first symptoms in patients with anti-NMDAR encephalitis are usually mental disorders and seizures, and patients with aphasia as the first symptom are rare. A 67-year-old male patient with NMDAR encephalitis who presented with hemianopsia, aphasia, and right hemiparesis as the first symptoms was reported.^[16]^ Two other case reports respectively described one 29-year-old female patient with anti-NMDAR encephalitis, both with motor aphasia as the first symptom onset.^[7,19]^ 2 case reports have described adolescent anti-NMDAR encephalitis patients with aphasia, both of which developed aphasia during the course of the disease.^[8,18]^ Interestingly, the majority of anti-NMDAR encephalitis patients who have been reported to present with aphasia have been female. Previously reported patients with aphasia as the first symptom were adults, and there were no reports of adolescents and children with anti-NMDAR encephalitis with aphasia as the first symptom. As far as we know, the current study is the first to report a case of adolescent anti-NMDAR encephalitis with aphasia as the first symptom, which broadens the range of clinical symptoms in anti-NMDAR encephalitis patients. In the future, anti-NMDAR encephalitis should be a disease to be considered in adolescent patients with aphasia as the first symptom.

Complicated peripheral nerve damage in patients with anti-NMDAR encephalitis is noteworthy.^[20]^ A study reported that a man who developed peripheral nerve damage following Guillain-Barre syndrome (GBS), then developed anti-NMDAR encephalitis.^[21]^ It has also been shown that patients with anti-NMDAR encephalitis due to paraneoplastic syndrome may have anti-Hu and anti-ganglioside antibodies in their bodies, which can also lead to peripheral nerve damage.^[22]^ However, the patient reported in our study had no tumor present, the peripheral nerve antibodies were negative as well, and there were no typical symptoms of GBS, which ruled out paraneoplastic syndrome and GBS. We considered that the etiology of the comorbid peripheral neuropathy in this case could be critical illness weakness (CIW). The clinical features of CIW were diffuse and symmetric muscle weakness after admission to the ICU, decreased tendon reflexes, and flaccid muscle tone, often involving the muscles of the extremities and respiratory muscles, with etiology related to multi-organ dysfunction and systemic inflammation.^[23–25]^ The patients reported in this study were consistent with critical illness, symmetrical muscle weakness, and flaccid muscle tone among the characteristics of CIW. Nevertheless, the patients did not present with respiratory muscle involvement, and the degree of recovery of the extremities was inconsistent and unexplained by CIW.

For tumor-free patients with anti-NMDAR encephalitis, immunotherapy is the mainstream treatment.^[26]^ Progressive therapy is commonly used to treat anti-NMDAR encephalitis in the clinic.^[27]^ Second-line therapies including rituximab and cyclophosphamide could be used when the efficacy of first-line therapies such as steroids, IVIG, and plasmapheresis is not satisfactory. For anti-NMDAR encephalitis refractory to first-line and second-line therapies, novel monoclonal antibodies may have the potential to become third-line therapy.^[28,29]^ Efgartigimod is a monoclonal antibody directed against FcRn, which is clinically used in the treatment of myasthenia gravis.^[30]^ Efgartigimod therapeutic mechanism is to prevent the recirculation of IgG by inhibiting the interaction of FcRn with IgG and leading to the elimination of unbound IgG,^[31]^ which is similar to the possible mechanism of IVIG and plasmapheresis for anti-NMDAR encephalitis.^[32]^ Therefore, when the patient’s symptom relief remained unsatisfactory after a prolonged course of first- and second-line therapy, we gave her Efgartigimod. After receiving 2 days of therapeutic doses of Efgartigimod, the patient’s muscle strength recovered from grade 3 to grade 5 in both lower limbs and from grade 3 to grade 4 in both proximal upper limbs. It is thought-provoking that whether the results would have been better if the patient had been able to receive Efgartigimod earlier. In conclusion, these results indicated that Efgartigimod may be a potential promising therapeutic option for anti-NMDAR encephalitis patients with no significant improvement after prolonged treatment, while its efficacy and safety remain to be validated in further clinical trials.

## 5. Conclusion

We report a case of an adolescent patient with anti-NMDAR encephalitis who had motor aphasia as the first symptom, which suggests that the possibility of anti-NMDAR encephalitis should be considered in adolescent with sudden motor aphasia in addition to adults. Meanwhile, the peripheral nerve axonal damage we reported in patients with anti-NMDAR encephalitis deserves attention with further studies. In addition, when the efficacy of both first-line and second-line treatments is unsatisfactory, Efgartigimod is an option to consider.

## Acknowledgments

We would like to thank the patient and families who provided the clinical information.

## Author contributions

**Conceptualization:** Niao Yang, Nao Yan.

**Data curation:** Lei Shen, Hanxing Liu, Xi Liu, Lei Zhang, Jin Wang.

**Funding acquisition:** Nao Yan.

**Writing – original draft:** Lei Shen.

**Writing – review & editing:** Niao Yang, Nao Yan.
